# St. Bartholomew's Hospital

**Published:** 1903-03-07

**Authors:** 


### The Hospital.

A Journal of

The Medical Sciences and Hospital Administration.

Vol. XXXIII.—No. 858. Edited by Sir Henry Burdett, K.C.B., and Solomon C. Smith, M.D., M.R.C.P. [March 7, 1903.

### St. Bartholomew's Hospital.

On the 21st ultimo under this heading we published a letter from "F.R.C.S.," in which he mentioned a rumour that instructions had been issued to the house surgeons and house physicians of St. Bartholomew's Hospital, by a responsible official of that institution, to immediately fill up all the vacant beds, if possible, and at the same time to select for admission as many patients as possible, from certain stated districts, which are in close vicinity to that hospital. We have now the highest authority to state that there is no truth whatever in this rumour, which is devoid of foundation. This is satisfactory, for the rumour was being freely circulated, and as we wish to help St. Bartholomew's Hospital to the fullest extent in this crisis of its history, we are glad to have been the means of eliciting this contradiction. The problem to be solved is surrounded with difficulties enough as it is, and will require the exercise of great tact and the united efforts of everybody concerned if the best solution is ever to be reached.

The grand hall of St. Bartholomew's Hospital contains a large number of very valuable portraits full of interest to the whole hospital world. Amongst them is a splendid representation of Sir William Lawrence, Bart., once Serjeant-Surgeon to the Queen, a member of the Imperial Institute of France, Professor of Anatomy and Surgery, and for many years a celebrated surgeon in London. He was the father of Sir Trevor Lawrence, the present treasurer of St. Bartholomew's, and it is pathetic to remember that he only lived two months to enjoy the baronetcy given him for his distinguished services. Sir William Lawrence was a man of considerable intellect and character, who exercised immense authority in his day and generation. That it should devolve upon the present treasurer to lead the policy to be followed, in the present momentous crisis of our oldest hospital, where the father spent his life and made his reputation, is a fact which may well encourage the hope, that full advantage will be taken of the present opportunity, which is certainly the greatest which has ever presented itself to the governors of any British hospital. Sir Trevor Lawrence has exhibited, under most trying circumstances, an excellent spirit and temper in all his public utterances and letters during the course of the current discussion on the affairs of St. Bartholomew's Hospital. He yields to no one of course in his interest in St. Bartholomew's Hospital, and will, we are confident, grudge neither the time nor effort required to steer the ship safely into port.

Apart from these considerations, in view of possible contingencies, we feel that every one who is actively interested in the hospitals might profitably pay a visit to St. Bartholomew's, and especially to the great hall, where the portraits are hung, because they could then realise in a way which it is impossible to realise otherwise, the splendid history of this great institution, with results which cannot fail to be highly beneficial to its welfare at the present juncture. If Sir Trevor Lawrence could arrange for the President to hold a reception of some of the oldest and most influential citizens of London in the grand hall, so soon as the Committee of Inquiry has completed its work and made its recommendations, we believe that nothing would be better calculated to secure the speedy subscription of the large sum, which in any circumstances it will probably be necessary for the governors to ask for from the citizens. Money will not be wanting if the scheme is wide enough to rebuild St. Bartholomew's Hospital on an eight acre site, and to place it upon a footing which will ensure its being worthy of our oldest hospital and medical school.

It is matter for congratulation that the medical staff should be unanimous in the conclusions at which they have arrived. That in itself augurs well for the future interests of St Bartholomew's, and should secure a satisfactory solution to the present difficulties. It is not easy of course for medical men to realise the enormous difficulties which confront the governors from the financial side. Finance is not of course the business of the medical staff, but it is certainly their business to take pains to understand the actual bearing which it has upon the present problem, and to bring their best brains to bear upon the consideration of the hygienic and medical aspects presented, so as to secure that nothing shall be done on their part to confuse the issue. That issue should be set forth with such clearness that any non-technical mind cannot fail to understand why certain suggestions and plans of reconstruction cannot be accepted as satisfactory, or even worthy of the past history of St. Bartholomew's Hospital. To-day it is possible to provide that St. Bartholomew's Hospital shall be saved from any risk, that its great position as a medical school will be maintained and extended by the adoption of a scheme of rebuilding, not only up to date, but in every way adequate to provide for the whole of the requirements formulated by the medical staff. Any plan to be acceptable must indicate how future requirements may be readily met without excessive cost, or the presence of difficultie s which the exercise of good management and forethought to-day can provide against.

